# Posterodorsal Medial Amygdala Regulation of Female Social Behavior: GABA versus Glutamate Projections

**DOI:** 10.1523/JNEUROSCI.1103-21.2021

**Published:** 2021-10-20

**Authors:** Caroline S. Johnson, Weizhe Hong, Paul E Micevych

**Affiliations:** ^1^Department of Neurobiology, David Geffen School of Medicine at University of California, Los Angeles, Los Angeles, California 90095; ^2^Laboratory of Neuroendocrinology of the Brain Research Institute, University of California, Los Angeles, Los Angeles, California 90095; ^3^Department of Biological Chemistry, David Geffen School of Medicine, University of California, Los Angeles, Los Angeles, California 90095

## Abstract

Social behaviors, including reproductive behaviors, often display sexual dimorphism. Lordosis, the measure of female sexual receptivity, is one of the most apparent sexually dimorphic reproductive behaviors. Lordosis is regulated by estrogen and progesterone (P4) acting within a hypothalamic-limbic circuit, consisting of the arcuate, medial preoptic, and ventromedial nuclei of the hypothalamus. Social cues are integrated into the circuit through the amygdala. The posterodorsal part of the medial amygdala (MeApd) is involved in sexually dimorphic social and reproductive behaviors, and sends projections to hypothalamic neuroendocrine regions. GABA from the MeApd appears to facilitate social behaviors, while glutamate may play the opposite role. To test these hypotheses, adult female vesicular GABA transporter (VGAT)-Cre and vesicular glutamate transporter 2 (VGluT2)-Cre mice were transfected with halorhodopsin (eNpHR)-expressing or channelrhodopsin-expressing adeno-associated viruses (AAVs), respectively, in the MeApd. The lordosis quotient (LQ) was measured following either photoinhibition of VGAT or photoexcitation of VGluT2 neurons, and brains were assessed for c-Fos immunohistochemistry (IHC). Photoinhibition of VGAT neurons in the MeApd decreased LQ, and decreased c-Fos expression within VGAT neurons, within the MeApd as a whole, and within the ventrolateral part of the ventromedial nucleus (VMHvl). Photoexcitation of VGluT2 neurons did not affect LQ, but did increase time spent self-grooming, and increased c-Fos expression within VGluT2 neurons in the MeApd. Neither condition altered c-Fos expression in the medial preoptic nucleus (MPN) or the arcuate nucleus (ARH). These data support a role for MeApd GABA in the facilitation of lordosis. Glutamate from the MeApd does not appear to be directly involved in the lordosis circuit, but appears to direct behavior away from social interactions.

**SIGNIFICANCE STATEMENT** Lordosis, the measure of female sexual receptivity, is a sexually dimorphic behavior regulated within a hypothalamic-limbic circuit. Social cues are integrated through the amygdala, and the posterodorsal part of the medial amygdala (MeApd) is involved in sexually dimorphic social and reproductive behaviors. Photoinhibition of GABAergic neurons in the MeApd inhibited lordosis, while photoactivation of glutamate neurons had no effect on lordosis, but increased self-grooming. These data support a role for MeApd GABA in the facilitation of social behaviors and MeApd glutamate projections in anti-social interactions.

## Introduction

Social behaviors are innate, adaptive, and necessary for the survival of the individual and the species. These behaviors are wide-ranging, including aggression, predator/prey responses, parenting, mating, and more (Choi et al., 2005; Stanley and Adolphs, 2013; Hong et al., 2014; Chen et al., 2019). Often, social behaviors display evident sexual dimorphism (Dulac and Kimchi, 2007; Li and Dulac, 2018; Chen et al., 2019), and this is true in reproductive behavior. Reproductive behavior is perhaps one of the most important social behaviors in the animal kingdom (Swanson, 2000). Therefore, the development and display of these behaviors is crucial for the reproductive fitness of a species, which has resulted in reproductive behaviors that are highly stereotypical and readily reproducible.

One of the most obvious sexually dimorphic reproductive social behaviors is lordosis. This measure of female sexual receptivity behavior is reflexive, and dependent on the coordination of internal and external cues. Lordosis manifests as an arching of the spine, elevation of the head, hindquarters, and tail to allow for intromission by the male (Beach, 1948; Pfaff et al., 1994). Lordosis is closely regulated by sex hormones to ensure that the display of the behavior occurs during the time in the cycle that maximizes reproductive success (Micevych and Ulibarri, 1992; Micevych et al., 2007). Lordosis is both an innate motivated behavior (Swanson, 2000) and a social behavior (Pfaff et al., 2008), and many neural circuits participate in its manifestation (Micevych and Meisel, 2017).

**Figure 1.**
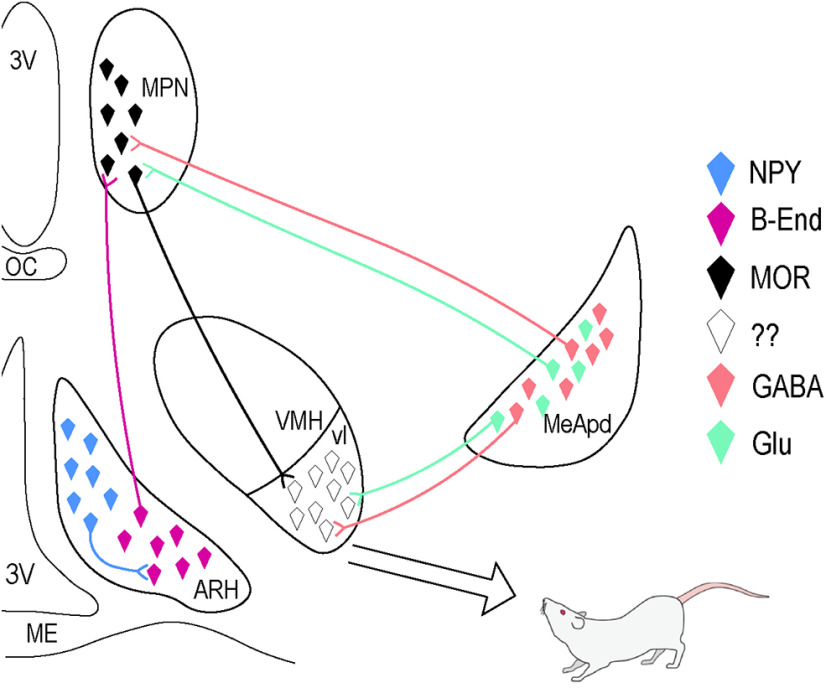
Hypothalamic-limbic lordosis circuit. Estradiol acts initially on neuropeptide Y (NPY) neurons in the ARH, which project to and activate POMC/β-endorphin (β-End) neurons. POMC/β-End neurons project to neurons containing μ-opioid receptor (MOR) in the MPN. The release of β-End onto MOR, a GPCR, activates and internalizes these receptors. This activation temporarily inhibits lordosis. Neurons from the MPN reach the VMHvl, the final integrative site in the hypothalamus. Inputs from posterodorsal part of the medial amygdala (MeApd) are GABAergic projection neurons, and also reach the VMHvl to integrate social information with the hormonal input and innervate the MPN. Glutamatergic neurons innervate the MPN and VMHvl as well. 3V, third ventricle; ME, median eminence; OC, optic chiasm. Adapted from Johnson et al. (2020).

The hypothalamic-limbic component is involved in the core expression of the behavior (Fig. 1; Micevych and Meisel, 2017). Although the entire circuit is sensitive to estradiol, the behavior requires sequential events in the arcuate nucleus (ARH), medial preoptic nucleus (MPN), and ventrolateral part of the ventromedial nucleus (VMHvl) of the hypothalamus, initiated by the activation of the estrogen receptor (ER)α in the ARH, before projections descend to the periaqueductal gray, hindbrain, and finally spinal motoneurons innervating musculature to produce the behavior (Pfaff, 1979). While the hypothalamic component largely regulates the hormonal aspect, the limbic component, including the amygdala, integrates social information.

Within the amygdala, the posterodorsal part of the medial amygdala (MeApd) is involved in a large number of social behaviors, many of which are sexually dimorphic, reflecting the well-documented sexual dimorphism of the region (Pfaff and Keiner, 1973; Hines et al., 1992; Canteras et al., 1995; Cooke et al., 1999; Cooke and Woolley, 2005). The MeApd is particularly associated with hypothalamic neuroendocrine regions, including those mediating reproductive behavior (Canteras et al., 1995).

The MeApd contains a high concentration of ERα (Pfaff and Keiner, 1973; Simerly et al., 1990), and GABA and glutamate neurons (Choi et al., 2005; Hong et al., 2014). GABA and glutamate modulate sexually dimorphic reproductive behaviors (Choi et al., 2005; Hong et al., 2014; Chen et al., 2019). Projections from here innervate the VMHvl (Canteras et al., 1995; Choi et al., 2005; Usunoff et al., 2009; Pardo-Bellver et al., 2012). MeApd GABA neurons contain vesicular GABA transporter (VGAT; McIntire et al., 1997). Glutamatergic neurons in the MeApd contain vesicular glutamate transporter 2 (VGluT2; Fremeau et al., 2001). In the MeApd, GABA appears to promote social behaviors, whereas glutamatergic neurons do not. While this circuit is largely the same in both sexes (Chen et al., 2019), differences begin to emerge at the cellular and molecular level, particularly within MeApd GABAergic neurons (Hong et al., 2014; Chen et al., 2019; Dalpian et al., 2019).

We hypothesized that both glutamatergic and GABAergic neurons in the MeApd modulate female reproductive behavior; GABA promotes lordosis while glutamate inhibits the behavior. To test these hypotheses, in the MeApd, halorhodopsin (eNpHR)-expressing adeno-associated viruses (AAVs) were inserted into VGAT-Cre neurons, and channelrhodopsin-expressing AAVs were inserted in VGluT2-Cre neurons. The effect of optogenetic photoinhibition or photoexcitation, respectively, on the expression of lordosis in sexually receptive, hormone-primed female mice was examined.

## Materials and Methods

### Animals

Adult [postnatal day (P)60] female VGAT-Cre (originally JAX #028862; Vong et al., 2011; The Jackson Laboratory) and VGluT2-Cre (originally JAX #28 863; Vong et al., 2011; The Jackson Laboratory) mice from our colony, and adult (P60) male C57BL/6J (The Jackson Laboratory, JAX #000664) were used for all experiments. Mice were group-housed two to a cage, on a 12/12 h light/dark cycle. Standard lab chow and water were provided *ad libitum*. All female mice were ovariectomized (ovx) and implanted with fiber optic cannulae (see below, Surgeries). Mice were randomly assigned to one of two groups per experiment:

#### Experiment 1

VGAT-Cre mice were assigned to either (1.1) eNpHR-AAV + hormone replacement + MeApd cannulae or (1.2) control AAV + hormone replacement + MeApd cannulae.

#### Experiment 2

VGluT2-Cre mice were assigned to either (2.1) channelrhodopsin (ChR2)-AAV + hormone replacement + MeApd cannulae or (2.2) control AAV + hormone replacement + MeApd cannulae.

See Table 1 for groups. All animal procedures were performed in accordance with the regulations of the University of California, Los Angeles Chancellor's Animal Research Committee.

**Table 1.** Groups included in both experiments, VGAT (experiment 1) and VGluT2 (experiment 2), including virus, hormone treatment, and *n*/group

Group	AAV	Hormone treatment	*n*
1.1	eNpHR	EB + P4	5
1.2	eYFP	EB + P4	5
2.1	Channelrhodopsin	EB + P4	7
2.2	tdTomato	EB + P4	5

EB, estrogen benzoate; P4, progesterone.

All hormones dissolved in safflower oil.

### Surgeries

Adult (P60) female mice were anesthetized under isoflurane and transfected with an AAV.

VGAT-Cre mice were transfected with an AAV expressing eNpHR (pAAV-double floxed-eNpHR-EYFP-WPRE-pA, Karl Deisseroth, Addgene plasmid #20949) or the control virus (pAAV-Ef1a-DIO EYFP, Karl Deisseroth, Addgene plasmid #27056). VGluT2-Cre mice were transfected with an AAV expressing ChR2 (AAV1.CAGGS. Flex.ChR2-tdTomato.WPRE.SV40; Addgene plasmid #18 917-AAV1) or the control virus (AAV1.CAG.Flex.tdTomato.WPRE.bGH; Addgene plasmid #28306-AAV1). In all mice, AAVs were delivered bilaterally into the MeApd (from bregma; AP: –1.50, ML: ±2.00, DV: –5.15) with a 5-µl Hamilton syringe (Hamilton Company, #7634-01) equipped with a 32-G removable needle (Hamilton Company, #7803-04), via World Precision Instruments Ultra-MicroPump (World Precision Instruments, UMP3-3), at a rate of 60 nl/min for a total volume of 100 nl per side. AAVs were allowed three weeks to incubate to allow for full expression before behavioral testing. Following injections, custom-made ferrule fiber cannulae (200-µm core diameter, 240-µm outer diameter, Doric Lenses) were implanted bilaterally immediately above the MeApd (from bregma; AP: −1.50, ML: ±2.00, DV: –5.05) and fixed on the skull with dental cement (Parkell, Metabond). Two weeks before behavioral testing, mice were bilaterally ovx, to allow sufficient time for the loss of endogenous hormones.

### Hormone replacement

All mice received subcutaneous injections of 17β-estradiol benzoate (EB) and progesterone (P4) dissolved in safflower oil, 2 h before lights out, over a 3-d period to mimic the estrous cycle. On days 1 and 2, mice received 20-µg EB, and on day 3, 500-µg P4.

### Behavioral optogenetic testing

For both experiments, each mouse was evaluated in a lordosis pre-test before optogenetic activation. Lordosis behavior was tested 2 h after lights off on the third day of hormone injections. See Johnson et al. (2020) for full testing procedures. Briefly, a single round of behavioral testing consisted of two interactions with a male mouse, each interaction lasting for 10 mounts by the male. Data from three sets of experimental behavioral tests were collected and averaged per test per animal. ∼15 min before behavior testing, sexually experienced adult male mice were placed in individual Plexiglas testing arenas. Immediately before placing the female in the arena with the male, the optogenetic patch cord (Doric Lenses) was attached to the implanted fiber optic cannula and remained attached for the duration of the behavior test. For each behavior test, the female was subject to a pre-test to determine sexual receptivity, measured by the lordosis quotient (LQ; the number of times a female displays lordosis/10 mounts by a male × 100). Mice that displayed LQ < 50 were considered unreceptive and testing was terminated for the day. Each test began when the female was placed in the cage and lasted until the male mounted the female 10 times. Following the pre-test, the female was removed from the arena and the laser was switched on for 2 min before being placed back in the arena with the male. Each mouse received both a pre-test and the optogenetic test once every 4 d, following the hormone replacement schedule in the previous section.

For experiment 1, photoinhibition of eNpHR (589 nm, continuous illumination, 5 s, 5–10 mW/mm^−2^) was applied for the duration of second interaction with the male. For experiment 2, photostimulation of ChR2 (473 nm, 20 Hz, 20-ms pulses,1–3 mW/mm^−2^) was applied for the duration of second interaction with the male. Behavior tests were recorded using a Yi Action Camera (XiaoYi Technology Co, LTD) and scored by observers blinded to the experimental condition. Following the final behavior test, mice were perfused 60 min after removal from the arena. For each animal, the results of the three pre-tests were averaged to a single LQ score, as were the results of the three optogenetic tests.

### Perfusion, brain removal, and sectioning

At the conclusion of behavioral testing, mice were transcardially perfused with cold 0.9% saline, followed by cold 4% paraformaldehyde (PFA) in Sorenson's buffer (pH 7.4) 60 min after removal from the testing arena. Brains were removed and postfixed in the same PFA solution for 24 h, then switched to 30% (w/v) sucrose in phosphate buffer for 2 d before being flash frozen in hexanes cooled on dry ice. Brains were sectioned 25 µm coronally using a Leica cryostat (Leica Biosystems, CM1950) and stored in a cryoprotectant solution at –20°C until used for immunohistochemistry (IHC).

### IHC

Tissue was processed for IHC as previously described (Johnson et al., 2020). Tissue sections containing the regions of interest (ROIs) were processed with GFP (experiment 1) or RFP (experiment 2), to augment fluorescent expression of the viral eYFP or tdTomato, respectively, and verify AAV expression in Cre-positive neurons. c-Fos IHC was used as proxy for neuronal activity. Sections were first washed in tris-buffered saline (TBS; pH 7.4) on a rotating table for 30 min at room temperature (RT) before being transferred to a blocking solution of 2% normal goat serum (NGS; Equitech-Bio, #SG30-0500) and 0.03% Triton X-100 (Sigma-Aldrich, #X100-100ML) in TBS for 1 h at RT. Sections were incubated in 2% NGS solution with the addition of rabbit anti-c-Fos and either mouse anti-GFP or guinea pig anti-RFP (Table 2) on a rotating table at 4°C for 48 h. Following incubation in the primary antibody solution, sections were again washed in TBS (3 × 10 min), and then placed in a solution containing the appropriate secondary antibody for each of the primary antibodies (Table 3). Sections were rinsed a final time in TBS and mounted onto SuperFrost slides (Fisher Scientific, #12-550-15). Once dry, slides were applied with mounting medium containing DAPI (DAPI Fluoromount-G, Southern Biotech, #0100–20) before being coverslipped and sealed with nail polish. Slides were stored in the dark at 4°C until imaging. See Tables 2, 3 for dilution and commercial source of antibodies used.

**Table 2.** Primary antibodies used in IHC, including commercial source and dilution of antibody

Primary antibody	Commercial source	Dilution
Mouse anti-GFP IgG_2A_	Invitrogen, A11120	1:500
Guinea pig anti-RFP	Synaptic Systems, 390004	1:10,000
Rabbit anti-c-Fos	Cell Signaling Technology, #2250	1:500

**Table 3.** Corresponding secondary antibodies used for IHC, including commercial source and dilution of antibody

Secondary antibody	Commercial source	Dilution
Alexa Fluor 488 goat anti-mouse IgG_2a_ (γ2a)	Invitrogen, A21131	1:2000
Alexa Fluor 594 goat anti-guinea pig IgG (H + L)	Invitrogen, A11076	1:2000
Alexa Fluor 594-conjugated goat anti-rabbit IgG (H + L)	Jackson ImmunoResearch, 111-585-144	1:2000

### Imaging

Images were obtained with a Zeiss LSM710 (Zen Blue Edition software, Zeiss) using the 405, 488, 561, and 594 laser lines, with appropriate emission filters to prevent optical bleed through. Sections containing the MeApd in both experiments were imaged with a 20× objective (Plan-APOCHROMAT 20×/0.8) for both 2D images, to confirm eNpHR or channelrhodopsin expression, and 3D images. In the *Z*-plane, the entire depth of the tissue was imaged. For experiment 2, the posteroventral region of the MeA (MeApv) was imaged as described as well. Sections containing the ARH and VMHvl were also imaged at 20×, in 2D only. Sections containing the MPN were imaged using a Leica Aperio VERSA Slide Scanner equipped with LAS X Life Science software suite (Leica Biosystems), using a 20× objective (HC PL APO 20×/0.8), resulting in a final optical magnification of 200×.

### Data analysis

Images were optimized by adjusting the brightness uniformly across all pixels. Images were analyzed using Imaris software (Imaris 9.2.1, Bitplane, Oxford Instruments Group) and ImageJ (Schneider et al., 2012). Colocalization of c-Fos with VGAT-Cre neurons (experiment 1) or with VGluT2-Cre neurons (experiment 2) was analyzed using the Colocalization and Surfaces modules in Imaris software. An ROI was drawn around the MeApd, using the Allen Brain Atlas Coronal Reference Atlas (Lein et al., 2007). The total number of cells in the MeApd expressing eYFP/GFP-immunoreactivity (-ir; experiment 1) or tdTomato/RFP-ir (experiment 2) only, c-Fos-ir only, and those expressing both labels were quantified. This was repeated in the MeApv in experiment 2. In Imaris, the volume of the ROI was quantified in µm^3^ as well, to ensure that the number of cells counted between groups could be reliably analyzed. For images in both experiments, c-Fos expression in ARH, VMHvl, and the medial part of the MPN (MPNm) was quantified using ImageJ, again using the Allen Brain Atlas Coronal Reference Atlas to delineate ROIs. The number of cells in each region expressing c-Fos-ir was analyzed using the Cell Counter plugin, and the area of the ROI was measured in µm^2^.

### Statistics

Statistics for behavioral and imaging data were analyzed using a Student's *t* test, Welch's *t* test, or two-way ANOVA followed by Tukey's multiple comparisons tests, as appropriate. All statistics were analyzed using GraphPad Prism version 9.0.2 for Windows (GraphPad Software). Significance for all analyses was set at *p* ≤ 0.05, and all values are expressed as mean ± SEM. Effect sizes were determined by Cohen's *d* test.

## Results

### AAV expression and fiber optic cannula placement

**Figure 2.**
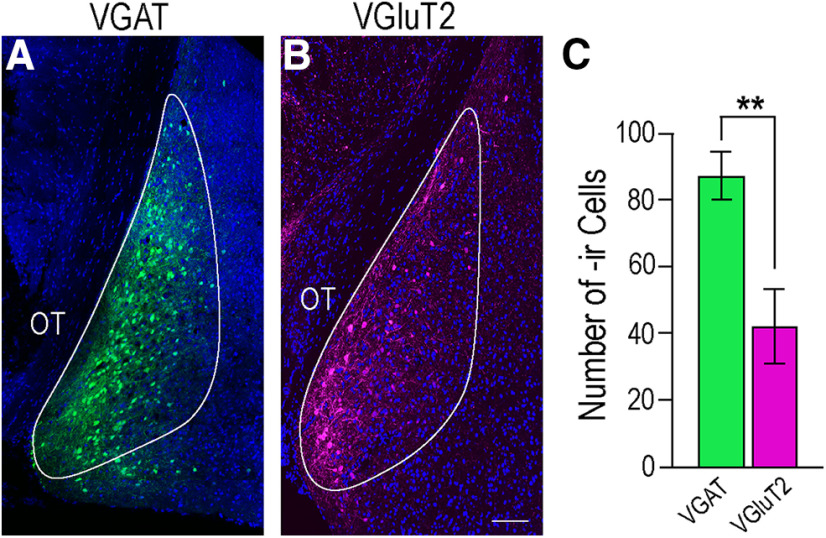
VGAT and VGluT2 expression in the MeApd. Cells expressing either VGAT (green, ***A***) or VGluT2 (magenta, ***B***) were found medially in the MeApd. DAPI counterstain is indicated in blue, the MeApd is outlined in white in each image. ***C***, More neurons expressed VGAT (87.20 ± 7.17, green bar) compared with VGluT2 (42.00 ± 11.22, magenta bar; *p* = 0.009). Values expressed as mean ± SEM; **p < 0.01. OT, optic tract. Scale bar: 100 µm.

**Figure 3.**
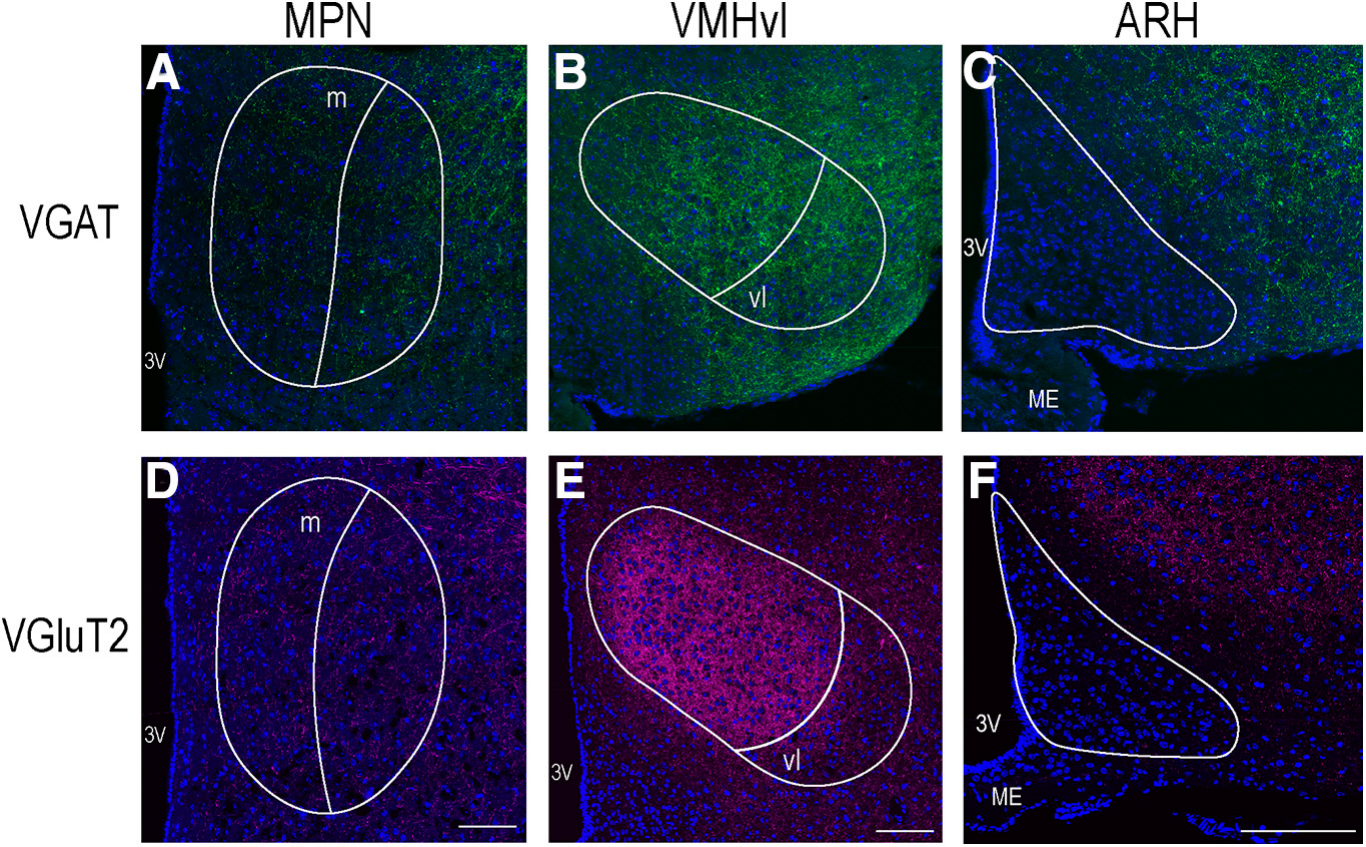
Innervation of reproductively relevant hypothalamic nuclei. Both GABAergic (green, ***A–C***) and glutamatergic (magenta, ***D–F***) fibers originating from the MeApd innervate the MPN (***A***, ***D***) and the VMHvl (***B***, ***E***). The ARH is largely devoid of either GABAergic (***C***) or glutamatergic (***F***) innervation from the MeApd. GABAergic innervation to the MPN and VMH is fairly equal between subdivisions of the nuclei (***A***, ***B***). Glutamate appears to innervate to the VMHvl to a lesser extent than the rest of the nucleus (***E***). DAPI counterstain in blue, neuroanatomical nuclei outlined in white. 3V, third ventricle; m, medial MPN; ME, median eminence; vl, ventromedial VMH. Scale bars: 100 µm.

eNpHR visualization was augmented using a primary antibody against GFP conjugated with Alexa Fluor 488 to verify the expression in VGAT-Cre cell bodies the MeApd, and channelrhodopsin (ChR2) was augmented using a primary antibody against RFP followed by Alexa Fluor 594 secondary antibody. Labeled GABAergic cell bodies were found spanning the entire dorsal-ventral extent of the region, in agreement with Bian (2013), while glutamatergic cell bodies were confined to the most medial portion of the same region (Fig. 2). Overall, significantly more VGAT-ir neurons (87.20 ± 7.17) than VGluT2-ir neurons (42.00 ± 11.22; *p* = 0.009; Student's *t* test) were visualized (Fig. 2). Fibers originating from MeApd VGAT neurons were found to innervate hypothalamic regions involved in reproduction, including the MPN and VMHvl (Fig. 3). Innervation from VGluT2 neurons was found in the MPN, but was qualitatively less apparent. Glutamatergic fibers were observed in the VMHvl, as well as other regions of the nucleus. No labeled fibers were visualized in the ARH of either set of animals (Fig. 3).

### Optogenetic inhibition of VGAT neurons in the MeApd attenuated sexual receptivity

**Figure 4.**
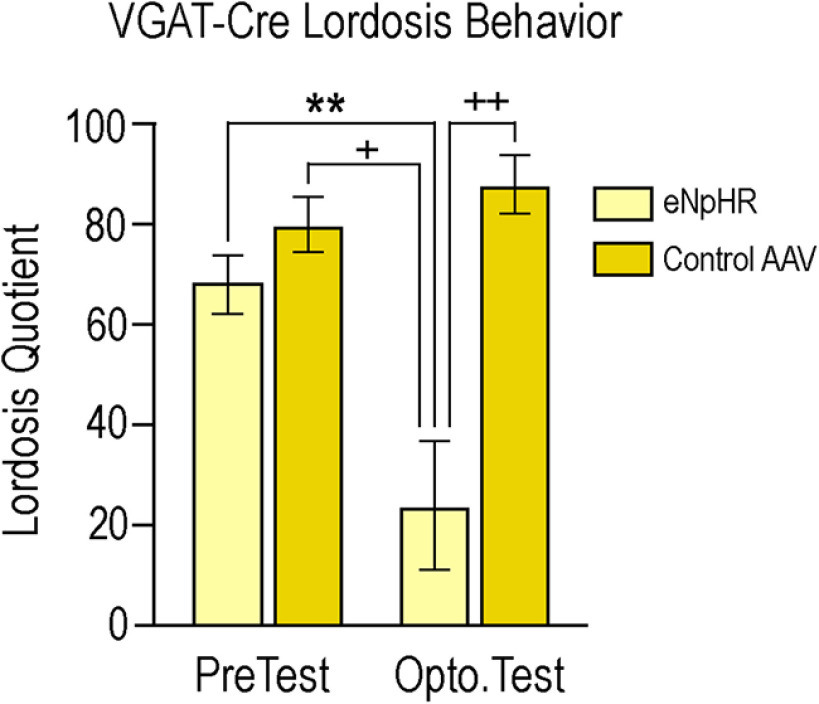
Photoinhibition of eNpHR-expressing VGAT neurons attenuates lordosis. Photoinhibition of eNpHR (light yellow bars) significantly reduced LQ as compared with the initial pre-test condition (*p* = 0.009), as well as the pre-test (*p* = 0.001) and photoinhibition condition (*p* = 0.0003) in mice that received the control virus (dark yellow bars). Two-way ANOVA followed by Tukey's multiple comparisons *post hoc* test indicated a significant interaction (*F*_(1,16)_ = 10.25, *p* = 0.006), and a main effect of viral treatment (*F*_(1,16)_ = 21.89, *p* = 0.0003) and photoinhibition (*F*_(1,16)_ = 4.911, *p* = 0.04). Values expressed as mean ± SEM; **p < 0.01, +*p* < 0.001, ++*p* < 0.0005.

A two-way ANOVA, followed by Tukey's multiple comparisons *post hoc* test, was conducted to analyze the effects of viral expression and photoinhibition on LQ (Fig. 4). Analysis indicated a significant interaction (*F*_(1,16)_ = 10.25, *p* = 0.006; effect size 0.89), as well as a main effect of viral treatment (*F*_(1,16)_ = 21.89, *p* = 0.0003; effect size 0.45) and photoinhibition (*F*_(1,16)_ = 4.911, *p* = 0.04; effect size 0.45).

No differences in mean LQ scores were observed between the two groups during the pre-test (group 1.1, 68.00 ± 5.83; group 1.2, 80.00 ± 5.48; *p* = 0.73), nor in mean LQ score between group 1.1 pre-test (68.00 ± 5.83) and group 1.2 photoinhibition test (88.00 ± 5.83; *p* = 0.34). Mice that expressed the control virus showed no difference in mean LQ score between the pre-test (LQ = 80.00 ± 5.47) and photoinhibition (LQ = 88.00 ± 5.83; *p* = 0.90).

In VGAT-Cre mice expressing eNpHR, photoinhibition significantly reduced mean LQ (24.00 ± 12.88) as compared with the pre-test (68.00 ± 5.83; *p* = 0.009). These mice also exhibited a photoinhibition-induced reduction in mean LQ compared with photoinhibition of the control group (88.00 ± 5.83; *p* = 0.0003). Finally, photoinhibition of group 1.1 also significantly reduced the mean LQ (24.00 ± 12.88) as compared with the pre-test of mice in group 1.2 (88.00 ± 5.83; *p* = 0.001).

### Optogenetic inhibition of VGAT neurons in the MeApd decreased c-Fos expression in the MeApd and VMHvl

Expression of c-Fos in the MeApd, and colocalization with VGAT-Cre neurons, was evaluated following photoinhibition. To ensure that the quantification of cells was comparable between the groups, the mean volume of the ROIs containing the MeApd was analyzed. There was no statistical difference in the size of the ROI between the two groups (*p* = 0.2). Furthermore, no difference was observed in the total number of Cre-positive cells quantified in the MeApd between groups (Student's *t* test, group 1, 76.6 ± 5.2; group 2, 87.2 ± 7.2; *p* = 0.3; data not shown).

**Figure 5.**
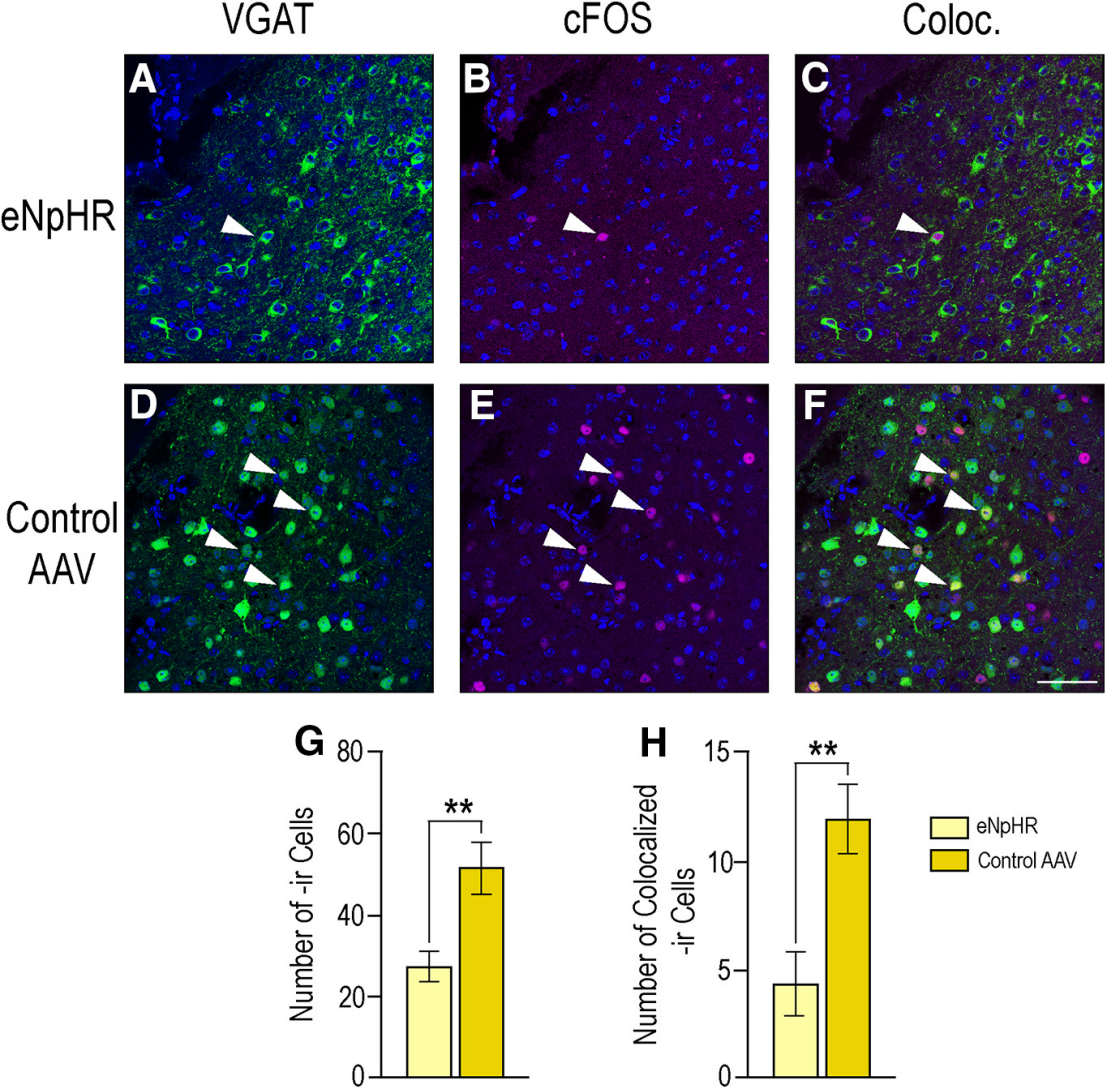
Photoinhibition of eNpHR-expressing VGAT neurons decreases overall c-Fos-ir and c-Fos colocalization with VGAT neurons in the MeApd. Photomicrographs in the top row show the expression of VGAT (***A***), c-Fos (***B***), and the merge of these two (Coloc., ***C***) in mice that received eNpHR. The bottom row shows the expression of VGAT (***D***), c-Fos (***E***), and merge of the two (Coloc., ***F***) in mice that received the control virus. VGAT-ir depicted in green, c-Fos-ir in magenta, DAPI counterstain in blue. Colocalization appears in yellow. Arrows indicate colocalization of immunoreactivity within specific cells in the MeApd. ***G***, Graph indicates that the total number of cells expressing c-Fos-ir was significantly attenuated in response to photoinhibition in mice that received eNpHR (27.4 ± 3.7; light yellow bars) compared with those mice that received the control virus (51.8 ± 6.4; dark yellow bars; *p* = 0.01). ***H***, Graph shows that colocalization of c-Fos and VGAT was also significantly attenuated in the MeApd in mice that received eNpHR (4.4 ± 1.5) as compared with those mice that received the control virus (12.0 ±1.6; *p* = 0.008). Values expressed as mean ± SEM; **p < 0.01. Scale bar: 25 µm.

**Figure 6.**
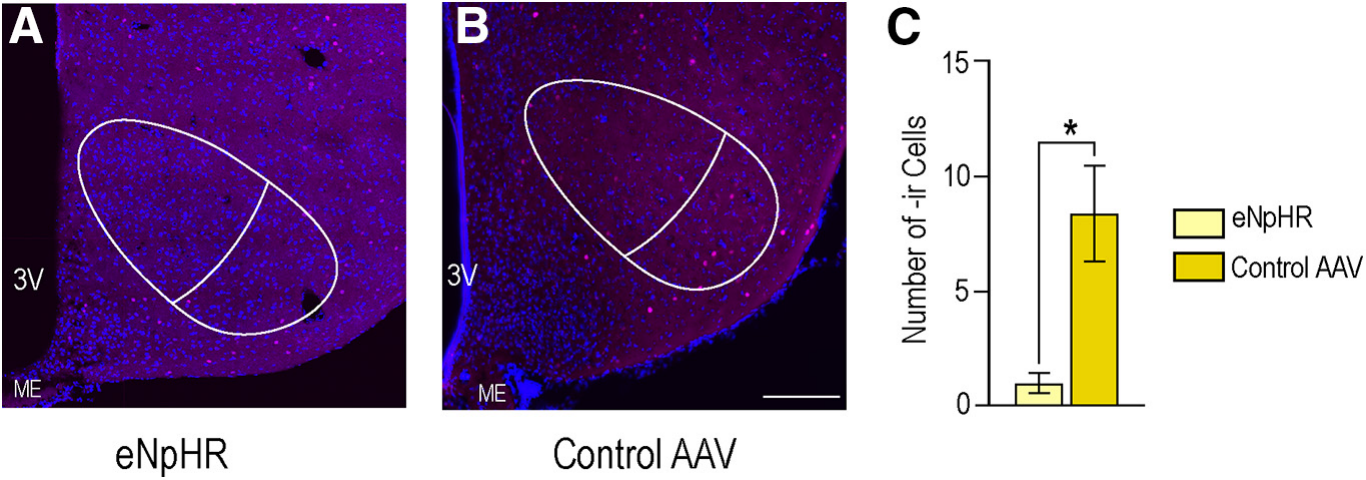
Photoinhibition of eNpHR-expressing VGAT neurons in the MeApd decreases c-Fos expression in the VMHvl. The number of cells expressing c-Fos-ir in the VMHvl was significantly reduced following photoinhibition of eNpHR (1.0 ± 0.4; ***A***) as compared with the control virus (8.4 ± 2.1; *p* = 0.02; ***B***). c-Fos-ir, magenta; DAPI, blue. Neuroanatomical outlines in white. ***C***, In graph, light yellow bars represent mice that received eNpHR, dark yellow bars represent mice that received the control virus. Values expressed as mean ± SEM; *p < 0.05. Scale bar: 100 µm.

In VGAT-Cre mice that received eNpHR, photoinhibition significantly decreased the total number of cells expressing c-Fos-ir within the MeApd (27.4 ± 3.7) when compared with those mice that received the control virus (51.8 ± 6.4; *p* = 0.01) determined by a Student's *t* test (Fig. 5). Furthermore, the number of cells expressing colocalization of VGAT-ir and c-Fos-ir was significantly reduced in mice that received eNpHR (4.4 ± 1.5) compared with those that received control virus following photoinhibition (12.0 ±1.6; *p* = 0.008; Student's *t* test; Fig. 5).

c-Fos-ir expression was analyzed in the VMHvl, as well. Again, the mean area of the VMHvl was compared between groups, and no difference was found (*p* = 0.9 Student's *t* test). Photoinhibition of eNpHR significantly reduced the number of cells expressing c-Fos-ir in the VMHvl (1.0 ± 0.4) compared with the control virus (8.4 ± 2.1; *p* = 0.02), determined by a two-tailed Welch's *t* test (*t*_(4.36)_ = 3.46, *p* = 0.01; Fig. 6). The number of cells expressing c-Fos-ir did not differ between groups in the ARH (group 1, 21.0 ± 6.9; group 2, 33.2 ± 2.7; *p* = 0.1) or the MPNm (group 1, 54.6 ± 11.8; group 2, 59.0 ±12.7; *p* = 0.8).

### Optogenetic excitation of VGluT2 neurons in the MeApd had no effect on the display of sexual receptivity

**Figure 7.**
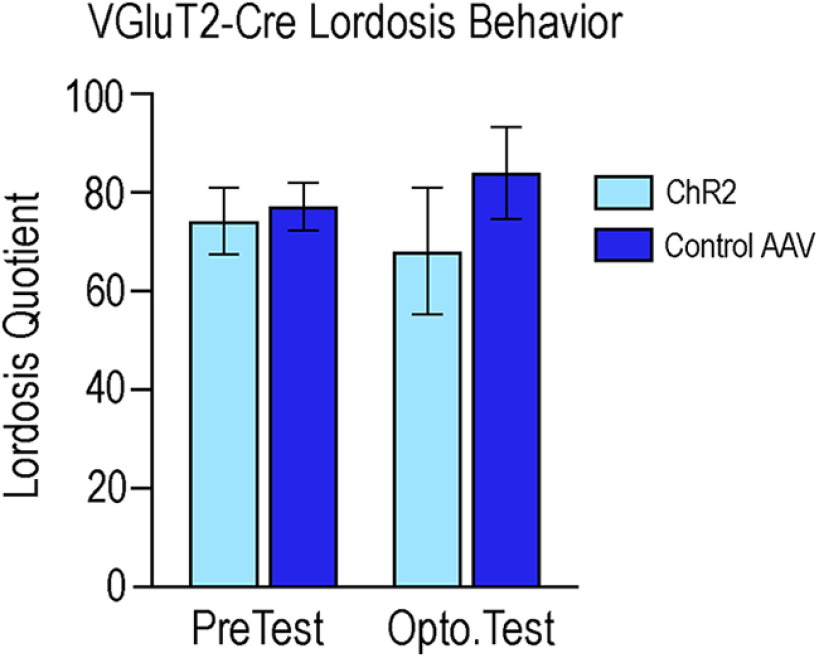
Photoexcitation of ChR2-expressing VGluT2 neurons did not affect lordosis behavior. No differences in LQ were detected in any condition, determined by a two-way ANOVA followed by Tukey's multiple comparisons *post hoc* test. Photoexcitation of ChR2-expressing VGluT2 neurons (light blue bars) in the MeApd did not result in differences between any condition, including no interaction (*F*_(1,20)_ = 0.445, *p* = 0.51), main effect of viral treatment (*F*_(1,20)_ = 0.936, *p* = 0.35), or photoexcitation (*F*_(1,20)_ = 0.001, *p* = 0.97). Mice that received the control virus are represented with dark blue bars.

A two-way ANOVA, followed by Tukey's multiple comparisons *post hoc* test, was also performed to analyze viral expression and photoexcitation on LQ (Fig. 7). Analysis indicated no differences between any condition: interaction (*F*_(1,20)_ = 0.445, *p* = 0.51), main effect of viral treatment (*F*_(1,20)_ = 0.936, *p* = 0.35), or photoexcitation (*F*_(1,20)_ = 0.001, *p* = 0.97).

### Optogenetic excitation of VGluT2 neurons in the MeApd decreased c-Fos expression in VGluT2 neurons in the MeApd

**Figure 8.**
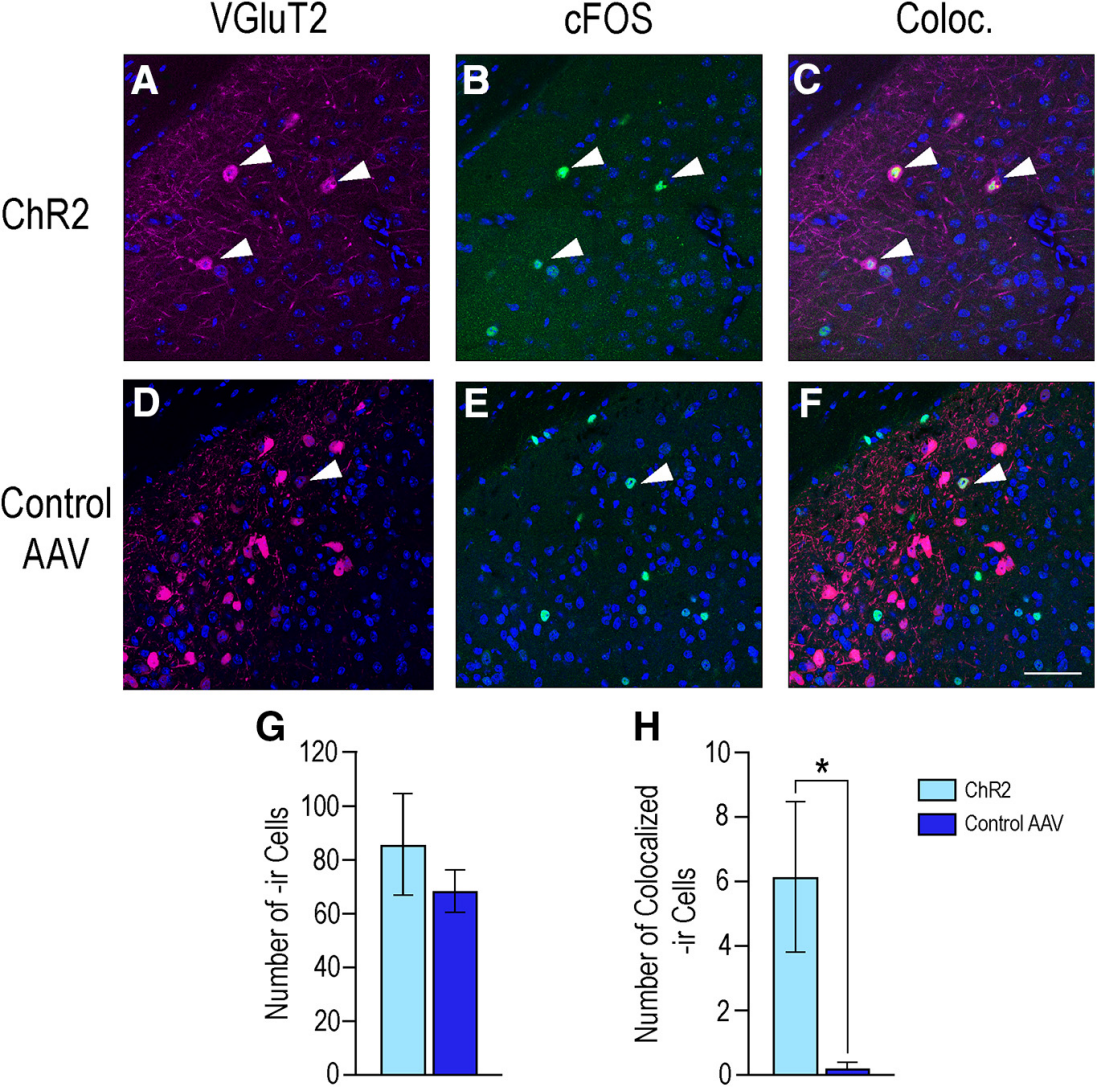
Photoexcitation of ChR2-expressing VGluT2 neurons did not alter overall c-Fos-ir but did reduce colocalization with VGluT2 neurons. Photomicrographs in the top row are of the expression of VGluT2 (***A***), c-Fos (***B***), and the colocalization of the two (Coloc., ***C***) in the MeApd in mice that received ChR2. The bottom row shows the expression of VGluT2 (***D***), c-Fos (***E***), and colocalization (Coloc., ***F***) in the MeApd of mice that received the control virus. VGluT2-ir is depicted in magenta, c-Fos-ir in green, DAPI counterstain in blue. Arrows indicate colocalization of immunoreactivity within specific cells in the MeApd. ***G***, Graph shows that there was no difference in total c-Fos expression between mice that received ChR2 (85.7 ± 18.9; light blue bars) and those that received the control virus (68.6 ± 8.07, *p* = 0.49; dark blue bars). However, photoexcitation of ChR2 (graph ***H***) resulted in an increase of c-Fos expression within VGluT2 neurons (6.14 ± 2.32) as compared with the control mice (0.20 ± 0.20; *p* = 0.04). Values expressed as mean ± SEM; *p < 0.05. Scale bar: 25 µm.

The expression of c-Fos, colocalization with VGluT2-Cre neurons, and volume of the MeApd were evaluated. No differences were found in volume between mice that received either virus, nor in the statistical difference in the number of neurons expressing the Cre-dependent fluorophore (Student's *t* test; data not shown). Similarly, no difference was detected in the total number of cells expressing c-Fos-ir between the control group (68.6 ± 8.07) and the group that received ChR2 (85.7 ± 18.9; *p* = 0.49, Student's *t* test; Fig. 8). However, control mice expressed significantly less colocalized cells (0.20 ± 0.20) as compared with mice with ChR2 (6.14 ± 2.32) as determined by the Welch's *t* test, which accounts for significantly different variance between the groups (*t*_(6.08)_ = 2.55, *p* = 0.04; Fig. 8).

As the MeApv is immediately adjacent to the MeApd and contains glutamatergic neurons that project to the VMHvl (Choi et al., 2005), c-Fos expression was analyzed in this region to ensure only MeApd VGluT2 neuronal activation occurred. There was no difference in number of VGluT2-labeled neurons (*p* = 0.12), overall c-Fos expression (*p* = 0.21), or colocalization in the MeApv between the two groups (*p* = 0.65; Student's *t* test; data not shown). Furthermore, histologic examination of cannula placement determined that the source of photoexcitation was not close enough to the MeApv to result in activity. Finally, optogenetic photoexcitation of VGluT2 neurons in the MeApd did not alter c-Fos expression in the MPN, VMHvl, or ARH compared with the control group (Student's *t* test; data not shown).

### All mice exhibited a progressive increase in LQ score with repeated sexual experience during the pre-test condition

In response to repeated sexual experience, mice and rats exhibit a progressive increase in LQ (Rajendren and Moss, 1993; McCarthy et al., 2017). To ensure that photomanipulation did not alter this pattern during subsequent pre-tests, we analyzed the LQs with one-way between-subjects ANOVAs to determine whether LQ score had the expected progression in each group (VGAT experiment: group 1, group 2; VGluT2 experiment: group 1, group 2). Two-way ANOVAs were used to test whether photomanipulation caused a difference in progressive LQ expression between groups 1 and 2, for each experiment.

**Figure 9.**
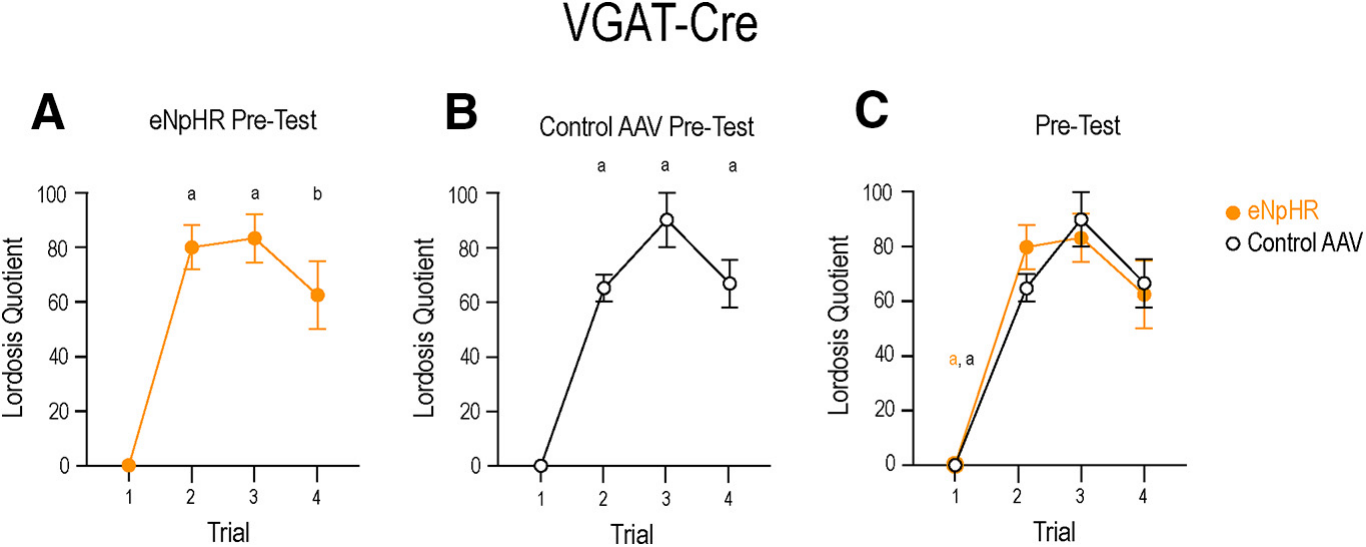
All VGAT-Cre mice displayed a progressive increase in LQ. Mice transfected with eNpHR (***A***) or the control virus (***B***) display a progressive increase in LQ score from the first sexual experience. A between-subjects one-way ANOVA indicated a significant effect of sexual experience on the LQ scores of mice that received eNpHR (*F*_(3,17)_ = 32.10; *p* < 0.0001). The LQ score of trial 1 was significantly lower than trials 2–4. There were no differences in LQ scores between trials 2–4. Similarly, repeated sexual experience resulted in an increase in LQ scores displayed by mice that received the control virus (*F*_(3,9)_ = 75.25; *p* < 0.0001). Again, trial 1 was significantly lower than trials 2–4, while there were no difference in LQ scores between trials 2, 3, and 4. ***C***, A two-way ANOVA indicated that trials 2–4 were not different between the groups. Trial 1 of the eNpHR group was significantly less than trial 2, 3, or 4 of both the eNpHR group and the control group (yellow a). Trial 1 of the control group was significantly less than trial 2, 3, or 4 of both the control group and the eNpHR group (black a). There was no difference between the first trial of either group. Yellow indicates eNpHR group, black indicates control AAV group. Data presented as mean ± SEM; a*p* < 0.0001, b*p* = 0.0001.

VGAT-cre mice infected with either eNpHR (Fig. 9*A*) or eYFP (Fig. 9*B*) each showed a progression in LQ scores during the pre-test condition. Mice that received the control AAV had the following LQ scores: trial 1, 0.00 ± 0.00; trial 2, 65.00 ± 5.00; trial 3, 90.00 ± 10.00; trial 4, 66.67 ± 8.82. A one-way ANOVA indicated a significant effect of sexual experience on LQ scores (*F*_(3,9)_ = 75.25; *p* < 0.0001). A *post hoc* multiple comparisons test indicated that trial 1 was significantly lower than trials 2 (*p* < 0.0001), 3 (*p* < 0.0001), and 4 (*p* < 0.0001). There were no difference in LQ scores between trials 2, 3, and 4.

Mice that received eNpHR-AAV exhibited the following LQ scores: trial 1, 0.00 ± 0.00; trial 2, 80.00 ± 8.17; trial 3, 83.33 ± 8.82; trial 4, 62.50 ± 12.50. A one-way ANOVA indicated a significant effect of sexual experience on LQ scores (*F*_(3,17)_ = 32.10; *p* < 0.0001). A *post hoc* multiple comparisons test indicated that trial 1 was significantly lower than trials 2 (*p* < 0.0001), 3 (*p* < 0.0001), and 4 (*p* = 0.0001). LQ scores were not statistically different between trials 2, 3, and 4.

To determine whether there were differences in LQ progression between groups (Fig. 9*C*), a two-way ANOVA was used, and indicated that there was no significant interaction (*F*_(3,28)_ = 0.75; *p* = 0.53) or main effect of AAV (*F*_(1,28)_ = 0.04; *p* = 0.84). A main effect of trial was revealed (*F*_(3,28)_ = 71.54; *p* < 0.0001). A *post hoc* multiple comparisons test indicated that in mice receiving eNpHR, trials 2–4 were significantly greater than their own trial 1, as well as trial 1 of mice that received the control virus. In mice that received the control virus, trials 2–4 were significantly greater than their own trial 1, and trial 1 of eNpHR mice (all *p*s < 0.0001). As before, there were no differences between trials 2, 3, and 4 between the groups (Fig. 9*C*).

**Figure 10.**
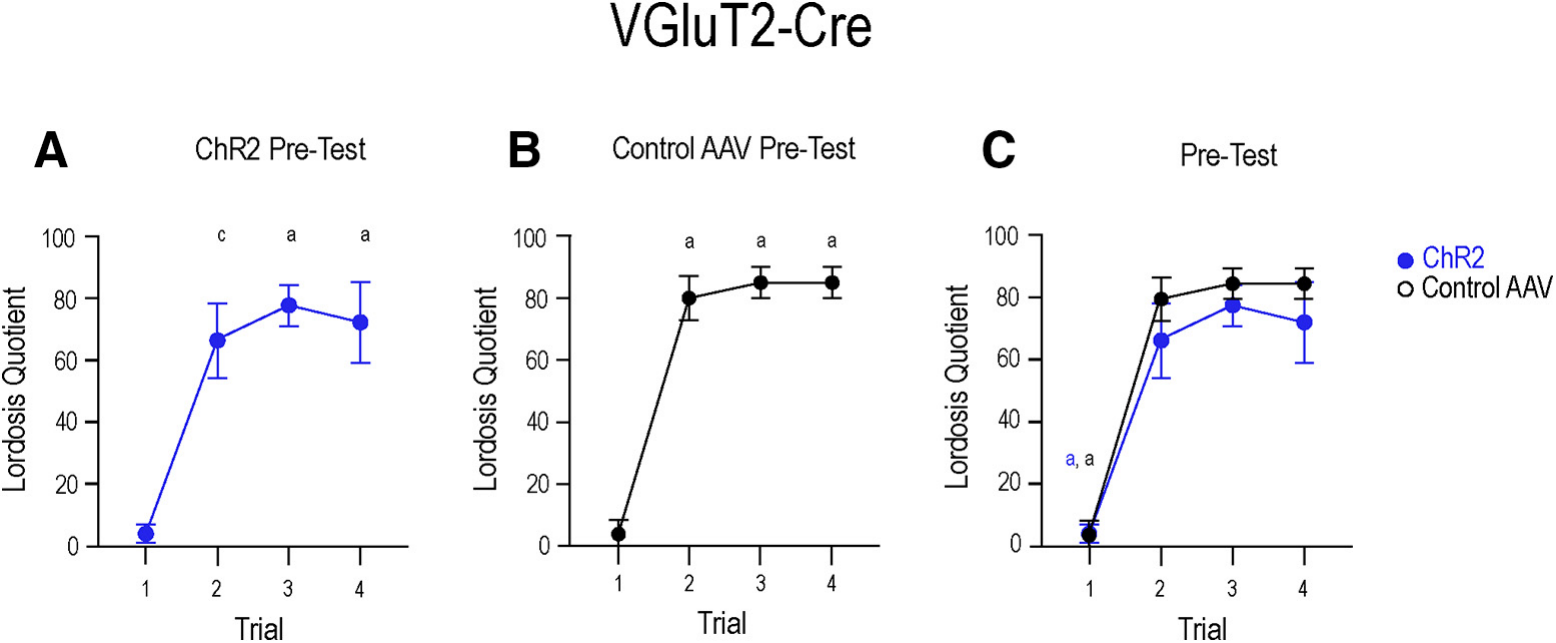
VGluT2-Cre mice displayed a progressive increase in LQ. Mice transfected with ChR2 (***A***) or the control virus (***B***) display a progressive increase in LQ score from the first sexual experience. A between-subjects one-way ANOVA indicated a significant effect of sexual experience on the LQ scores of mice that received ChR2 (*F*_(3,14)_ = 23.42; *p* < 0.0001). As seen with the VGAT experiment, the LQ score displayed in trial 1 was significantly lower than trials 2–4. There were no differences in LQ scores between trials 2, 3, and 4. Again, repeated sexual experience resulted in an increase in LQ scores in mice that received the control virus (*F*_(3,11)_ = 60.24; *p* < 0.0001). Trial 1 was significantly lower than trials 2–4, while there were no difference in LQ scores between trials 2, 3, and 4. ***C***, A two-way ANOVA indicated that trials 2–4 were not different between the groups. Mice that received ChR2 displayed significantly lower LQ scores in trial 1 than in trial 2, 3, or 4 of both the ChR2 group and the control group (blue a). Trial 1 of the control group was significantly less than trial 2, 3, or 4 of both the control group and the ChR2 group (black a). There was no difference between the first trial of either group. Blue indicates ChR2 group, black indicates control AAV group. Data presented as mean ± SEM; a*p* < 0.0001, c*p* = 0.0005.

VGluT2-cre mice infected with either channelrhodopsin (Fig. 10*A*) or tdTomato (Fig. 10*B*) each also showed a progression in LQ scores during the pre-test condition. Mice that received the control AAV displayed the following LQ scores: trial 1, 4.00 ± 4.00; trial 2, 80.00 ± 7.07; trial 3, 85.00 ± 5.00; trial 4, 85.00 ± 5.00. A significant effect of sexual experience on LQ scores was noted (*F*_(3,11)_ = 60.24; *p* < 0.0001 one-way ANOVA). A *post hoc* multiple comparisons test indicated that trial 1 was significantly lower than trials 2 (*p* < 0.0001), 3 (*p* < 0.0001), and 4 (*p* < 0.0001), and there were no differences in LQ scores between trials 2, 3, and 4.

Mice that received ChR2-AAV displayed the following LQ scores: trial 1, 3.33 ± 2.11; trial 2, 66.67 ± 12.02; trial 3, 78.00 ± 6.63; trial 4, 72.50 ± 13.15. As before, a significant effect of sexual experience on LQ scores was noted (*F*_(3,14)_ = 23.42; *p* < 0.0001, one-way ANOVA). Trial 1 was significantly lower than trials 2 (*p*= 0.0005), 3 (*p* < 0.0001), and 4 (*p* < 0.0001; *post hoc* multiple comparisons test). LQ scores plateaued and were statistically similar between trials 2, 3, and 4.

As in the VGAT experiment, two-way ANOVA analysis indicated neither a significant interaction (*F*_(3,25)_ = 0.35; *p* = 0.79) or a main effect of AAV (*F*_(1,25)_ = 2.50; *p* = 0.13) on LQ progression (Fig. 10*C*). Similarly, there was a main effect of trial (*F*_(3,25)_ = 64.03; *p* < 0.0001). Also similar to the VGAT experiment, a *post hoc* multiple comparisons test indicated that in mice that received ChR2, trials 2–4 were significantly greater than their own trial 1, as well as trial 1 of mice that received the control virus. In mice that received the control virus, trials 2–4 were significantly greater than their own trial 1, and trial 1 of mice that received ChR2 (all *p*s < 0.0001). As with the VGAT experiment, there were no differences between trials 2, 3, and 4 between the groups (Fig. 10*C*).

### Optogenetic photoexcitation of VGluT2 neurons in the MeApd increased self-grooming

**Figure 11.**
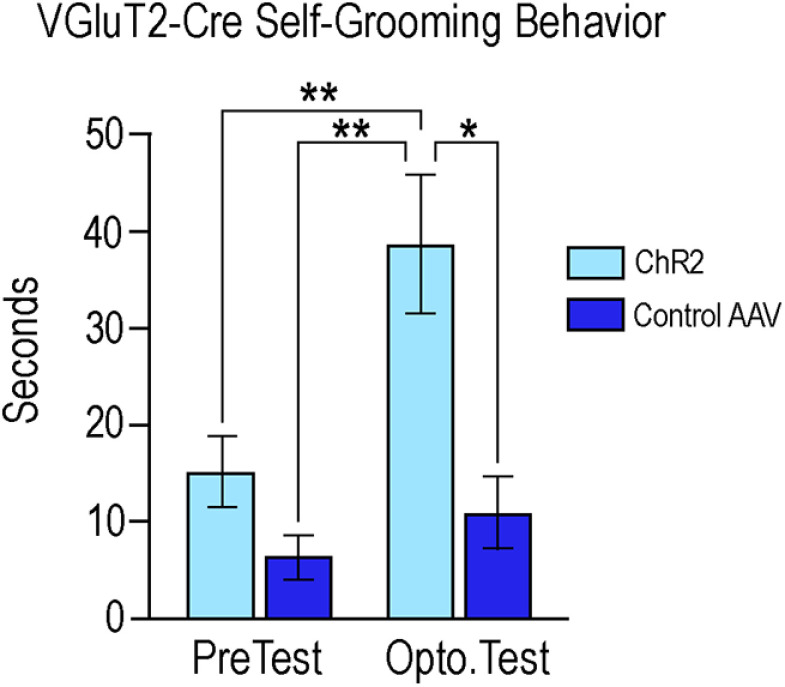
Photoexcitation of ChR2-expressing VGluT2 neurons promotes self-grooming. Photoexcitation of ChR2-expressing VGluT2 (light blue bars) neurons significantly increased the total time in seconds that mice spent self-grooming (38.62 ± 7.20) as compared with the pre-test condition (15.10 ± 3.67; *p* = 0.006), as well as to both the pre-test (6.54 ± 2.12; *p* = 0.003) and photoexcitation test (10.92 ± 3.80; *p* = 0.01) of the control group (dark blue bars). A two-way ANOVA followed by Tukey's multiple comparisons *post hoc* test indicated no significant interaction (*F*_(1,80)_ = 2.33, *p* = 0.13), but a main effect of viral treatment (*F*_(1,80)_ = 8.54, *p* = 0.005) and photoexcitation (*F*_(1,80)_ = 5.02, *p* = 0.02). Values expressed as mean ± SEM; *p < 0.05, **p < 0.01.

Photoexcitation of VGluT2 neurons in the MeApd increases self-grooming in males (Hong et al., 2014). In the present study, a two-way ANOVA, followed by Tukey's multiple comparisons *post hoc* test, compared the effects of viral expression and photoexcitation on self-grooming behavior in females (Fig. 11). No significant interaction (*F*_(1,80)_ = 2.33, *p* = 0.13) was noted, but significant effects of viral treatment (*F*_(1,80)_ = 8.54, *p* = 0.005) and photoexcitation (*F*_(1,80)_ = 5.02, *p* = 0.02) were apparent.

In VGluT2-Cre mice that received ChR2, photoexcitation significantly increased the total time that mice spent self-grooming (38.62 ± 7.20 s) compared with the pre-test condition (15.10 ± 3.67 s; *p* = 0.006), as well as to both the pre-test (6.54 ± 2.12 s; *p* = 0.003) and photoexcitation test (10.92 ± 3.80 s; *p* = 0.01) of the control group.

During the pre-tests, total time spent self-grooming between the ChR2 mice (15.10 ± 3.67 s) and control mice (6.54 ± 2.12 s;) was not statistically different (*p* = 0.76). Similarly, no difference was found between ChR2 mice pre-test (15.10 ± 3.67) and the photoexcitation test of control mice (10.92 ± 3.80; *p* = 0.96). Finally, control mice showed no difference in total time spent grooming between the pre-test (6.54 ± 2.12 s) and photoexcitation test (10.92 ± 3.80 s; *p* = 0.97).

### More cells showed c-Fos colocalization with VGAT- than with VGluT2-neurons in the MeApd of sexually receptive mice

**Figure 12.**
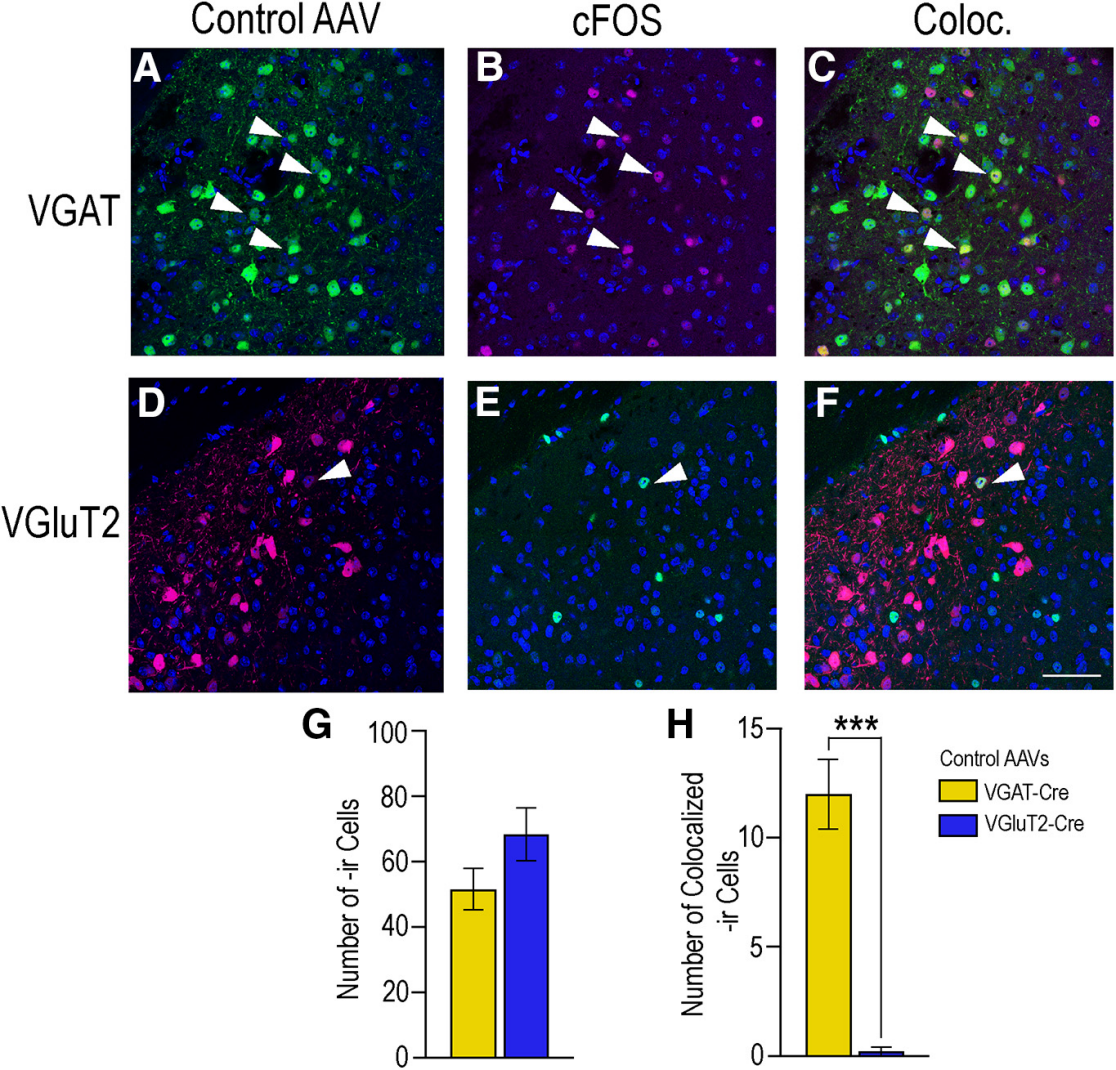
Activation of VGAT and VGluT2 neurons in the MeApd during sexual receptivity. Images in the top row show VGAT (green, ***A***), c-Fos (magenta, ***B***), and the colocalization of the two (***C***) in the MeApd. Images in the bottom row show VGluT2 (magenta, ***D***), c-Fos (green, ***E***), and colocalization (***F***). In sexually receptive control mice, c-Fos expression in the MeApd was not different between control VGAT-Cre mice (51.80 ± 6.35; ***B***) and control VGluT2-Cre mice (68.60 ± 8.07; ***D***). In these control mice, c-Fos colocalization was significantly greater in VGAT-Cre mice (12.00 ± 1.61; ***G***) than in VGluT2-Cre mice (0.20 ± 0.20, *p* = 0.002; ***H***). DAPI counterstain in blue. Arrows indicate colocalization of immunoreactivity within cells in the MeApd. Yellow bars represent VGAT-Cre, blue bars represent VGluT2-Cre. Values expressed as mean ± SEM; ***p < 0.005. Scale bar: 25 µm.

Mice that received either control virus remained sexually receptive (Figs. 4, 7). In the MeApd, a similar number of c-Fos-ir cells were visualized in the VGAT (51.80 ± 6.35) and VGluT2 control groups (68.60 ± 8.07; *p* = 0.14; Student's *t* test; Fig. 12). However, in these sexually receptive mice, significantly more cells had c-Fos colocalized with VGAT neurons (12.00 ± 1.61) compared with VGluT2 neurons (0.20 ± 0.20, *p* = 0.002), determined by a Welch's *t* test (*t*_(4.33)_ = 1.94, *p* = 0.001). The number of cells c-Fos-ir did not differ statistically between the two sets of control mice in the MPN (*p* = 0.95), VMHvl (*p* = 0.07), or ARC (*p* = 0.20; data not shown, Student's *t* test).

## Discussion

The major finding of these experiments is that photoinhibition of GABA neurons in the female mouse MeApd significantly attenuated lordosis behavior compared with control mice (Fig. 4), indicating that activity in GABAergic neurons in the MeApd was involved in the display of lordosis behavior. However, photoexcitation of MeApd glutamatergic neurons in mice did not affect the expression of lordosis (Fig. 7), but did increase the time the mice spent self-grooming (Fig. 11), suggesting that glutamatergic activity in the region directs behavior away from social interactions. These results are congruent with the role of the amygdala as a gate-keeper for many social functions including reproduction. Of particular importance in reproduction is the MeApd, with its populations of GABAergic and glutamatergic neurons (Fig. 2; Choi et al., 2005; Bian et al., 2008; Hong et al., 2014; Chen et al., 2019).

Of these two, GABAergic neurons predominate, and send projections to downstream regions involved in diverse aspects of reproductive behavior (Choi et al., 2005). Many of these behaviors are sexually dimorphic, and GABA plays a role in the expression of these behaviors (McCarthy et al., 1990; Luine et al., 1999; Hong et al., 2014; Chen et al., 2019), while glutamate from the MeApd appears to inhibit social behaviors, including reproductive behaviors (Hong et al., 2014; Chen et al., 2019). Furthermore, a molecular study investigating sex differences in GABAergic and glutamatergic neurons in the MeApd found that while there are a significant number of molecular differences within GABAergic neurons between the sexes, the differences within glutamatergic neurons are minimal (Chen et al., 2019). Therefore, we hypothesized that GABAergic neurons in the MeApd play a role in facilitating lordosis, while glutamatergic neurons are involved in the inhibition of the behavior.

We found that in both VGAT-Cre and VGluT2-Cre mice, neurons expressing the injected AAV were found in the medial aspect of the MeApd. VGAT neurons extended the entire dorsal-ventral length along the border of the optic tract, while VGluT2 neurons were more circumscribed in the same region (Fig. 2). This is in agreement with previous findings that also demonstrated that the majority of the MeApd neurons projecting to other reproductively relevant regions in the hypothalamus, including the VMHvl, are GABAergic as rather than glutamatergic (Choi et al., 2005; Bian et al., 2008; Hong et al., 2014; Chen et al., 2019). This GABAergic population expresses the transcription factor Lhx6, and appears to be activated specifically by reproductive cues. Glutamatergic neurons in the MeApd do not express Lhx6 (Choi et al., 2005).

Similarly, we observed the expected pattern of eYFP-labeled fibers in the hypothalamus; innervating the MPNm and the VMHvl, but avoiding the VMHdm (Fig. 3). In agreement with previous studies, the eYFP GABA fibers largely avoided the ARH (Canteras et al., 1995; Choi et al., 2005; Bian et al., 2008). The pattern of tdTomato-labeled fibers in VGluT2-Cre mice largely mirrored that of the eYFP-labeled projections seen in the VGAT-Cre mice, although innervation of the VMHvl was qualitatively lighter (Fig. 3). Glutamatergic neurons in the MeApd do not significantly innervate the VMHvl, but MeApv glutamate fibers do (Choi et al., 2005). We expect that the sparse innervation of the VMHvl may reflect AAV expression from neurons along the border of the two regions.

Optogenetic photoinhibition of MeApd GABAergic neurons significantly attenuated the expression of lordosis in sexually receptive female mice (Fig. 4). The contribution of the MeA in lordosis is in line with previous studies showing that lesioning the MeA decreases LQ (Rajendren and Moss, 1993; DiBenedictis et al., 2012). These lesions also abolished the progressive increase in LQ that normally occurs in response to repeated sexual experience (Rajendren and Moss, 1993), and this finding has been replicated using designer receptors activated exclusively by designer drugs (DREADDs) to silence MeA neurons, inhibiting lordosis (McCarthy et al., 2017). Following several trials of DREADD-induced neuronal silencing, mice given saline rather than clozapine-N-oxide, the ligand for DREADDs, exhibited an LQ indistinguishable from that of the control group (McCarthy et al., 2017), indicating that MeA neurons are involved in the acute expression of lordosis. In the present study, we observed a similar phenomenon: there was no difference in pre-test LQs (given to all mice) between the groups as testing progressed. This indicates that neither the acute inhibition of GABA neurons (Fig. 9) nor the excitation of glutamatergic neurons (Fig. 10) affected long-term expression of lordosis. The present study demonstrated that GABAergic MeApd neurons, specifically, are crucial for the expression of lordosis, and refines our understanding of the cell types in the MeA involved in this particular neurocircuit. While an interesting confirmatory study would be to stimulate lordosis in a mouse primed with a subthreshold dose of estradiol, these experiments are not as easily done in mice as rats, which have more linear and reproducible response to estradiol priming.

In our study, photoinhibition of GABAergic neurons in the MeApd significantly decreased c-Fos expression in the region, as well as within GABAergic neurons (Fig. 5). Given that c-Fos expression is a marker of neuronal activity (Bullitt, 1990), this decrease indicates that: (1) photoinhibition in this region did inhibit neuronal activity, and (2) this inhibition of activity, including within GABAergic neurons, occurred concurrently with an inhibition of behavior.

Photoinhibition of GABAergic neurons in the MeApd decreased c-Fos expression in the VMHvl (Fig. 6). The VMHvl is required for lordosis behavior. While the VMHvl ERα neurons are largely glutamatergic (Hashikawa et al., 2017), the majority of these neurons do not express c-Fos in response to mating (Calizo and Flanagan-Cato, 2003). GABAergic neurons in the VMHvl are interneurons (Jang et al., 2001), and GABA in the VMHvl facilitates reproductive behavior (McCarthy et al., 1990; Luine et al., 1999). It seems plausible then that the decrease in c-Fos expression in the VMHvl after MeApd VGAT photoinhibition reflects a decrease in GABAergic interneuron activity.

Photoexcitation of ChR2-expressing VGluT2 neurons did not significantly affect lordosis (Fig. 7), but did increase self-grooming behavior (Fig. 11). A MeApd glutamate-facilitated increase in self-grooming is consistent with previous findings (Hong et al., 2014). The present study confirms behaviorally that the activation of MeApd glutamate neurons in females also suppressed social behaviors and increased self-grooming. Self-grooming has been used as a mouse model for autism spectrum disorders (ASD), and these anti-social behaviors are correlated with activity in glutamate neurons in the amygdala (Etherton et al., 2009; Blundell et al., 2010; Silverman et al., 2010). On the other hand, activity of amygdala GABA neurons suppresses anti-social behavior, and has lent evidence to the “excitation/inhibition imbalance hypothesis” in ASD (Rubenstein and Merzenich, 2003).

We found that photoexcitation of VGluT2 neurons did not change the total number of neurons expressing c-Fos in the MeApd, but did increase the colocalization of c-Fos with VGluT2 as compared with control mice (Fig. 8). The lack of difference in overall MeApd c-Fos expression may be because of significantly less VGluT2 neurons in the region overall (Fig. 2). Alternatively, this may suggest that activated VGluT2 neurons interact with other, here uncharacterized, neuronal populations within the MeApd to orchestrate behavioral output. Additionally, photoexcitation of VGluT2 neurons did not alter c-Fos expression in the VMHvl compared with control mice, a result not unexpected given the neuroanatomical projection patterns of the MeApd glutaminergic neurons (Choi et al., 2005).

Finally, we compared c-Fos expression between GABAergic and glutamatergic neurons in control mice. We found significantly more VGAT than VGluT2 neurons in the MeApd (Fig. 2). Moreover, sexually receptive female mice display significantly more c-Fos expression in VGAT than in VGluT2 neurons (Fig. 12). Again, this finding supports a role for GABAergic activity in social behaviors, and lends further evidence that MeApd glutamatergic activity is not involved in promoting these behaviors.

We applied photoinhibition or photoexcitation to appropriately transfected cell bodies in the MeApd, a region with many known downstream targets (Canteras et al., 1995; Choi et al., 2005; Pardo-Bellver et al., 2012). The change in cell body activity in the MeApd may have affected regions not explicitly addressed in this study, i.e., the bed nuclei of the stria terminalis, the ventral premammillary nucleus, etc., which will have in turn affected behavior. Future experiments will include optogenetic manipulation of MeApd terminals in the above-mentioned regions, as well as the VMHvl. However, given that lordosis behavior decreased following photoinhibition of GABAergic neurons, suggests that the MeApd does act as a gate-keeper for this behavior-integrating sensory and hormonal cues from upstream, and orchestrating activation downstream regions through specific neurons.
